# Effects of the Participatory Action Research on Reducing the Risk of Metabolic Syndrome in Adult Women

**DOI:** 10.3390/ijerph182111103

**Published:** 2021-10-22

**Authors:** Yong-Sook Eo

**Affiliations:** College of Nursing, Dongguk University, Gyeongju 38066, Korea; nursingeo@dongguk.ac.kr; Tel.: +82-54-770-2621

**Keywords:** participatory action research, metabolic syndrome, adult women, psychosocial factors, health behavior

## Abstract

This study aimed to evaluate the effects of a participatory action research (PAR) on reducing the metabolic syndrome risk factors among the Koran adult women. The effectiveness of the PAR intervention was examined using a one-group pretest-post-test design. The data were collected from 58 adult women living in a community health center in Ulsan, Korea, between May and November 2016. The psychosocial factors (empowerment, social support, and quality of life), metabolic-related indices, and health behaviors were collected to measure the intervention’s efficiency. After applying it, the participants’ empowerment, social support, and health-related quality of life increased significantly, as compared to the pre-test. Furthermore, their metabolic-related indices improved significantly in the post-test, as compared to the pre-test. Therefore, the PAR intervention was found to be effective in enhancing the psychosocial factors, metabolic-related indices, and health behaviors in the aforementioned population and could be applied to other community health centers.

## 1. Introduction

### 1.1. Necessity for the Study

The prevalence rate of metabolic syndrome (MetS) among Korean adults aged 19 years and older is 26.2% in 2016 [1], with 72.2% of them affected by one or more of its risk factors [2]. As compared to the men in their 30s, the pattern of MetS in women is characterized by a decreased incidence and a drastic increase after the age of 50 years [2], which is ascribable to the menopause-related reduction in the female hormone levels, leading to abdominal obesity [3]. MetS elevates not only the risk of cardiovascular diseases by more than four times [4], but also that of chronic illnesses such as diabetes, hypertension, stroke, and even cancer, leading to death [5]. Therefore, a preventive intervention focusing on adult women with MetS risk factors would lower MetS and the prevalence of the related chronic diseases.

The factors associated with MetS in adult women are multi-level, ranging from individual to group to organizational stages. The individual-level factors include lifestyle habits, such as diet, exercise, weight control, body mass index (BMI) reduction, smoking cessation [4], and cognitive aspects, including health perception, exercise efficacy, and perceived intensity [6]. At the group level, social support can be expected from family, friends, and colleagues [7]. It is essential to organize group meetings with peers experiencing similar problems to support one another during a MetS intervention requiring continuous lifestyle improvements [3,7]. Furthermore, at the organizational and community levels, the community-based participatory approach effectively provides incessant motivation for voluntary participation and maintenance of healthy behaviors [3,8].

As discussed previously, interventions for conditions requiring lifestyle modification, such as the MetS, need to be developed for various relevant factors and patients’ lives. One such intervention approach is the participatory action research (PAR) [8]. It enables individuals to consider multi-level dynamics and connection, relationship, feedback, and interactions among them [9]. Additionally, it requires the research participants to identify and resolve the problem of interest by generating practical, helpful knowledge adapted to their lives [9,10]. Action research is a process of inquiry in which participants proactively explore their personal and social lives to continuously enhance them. The PAR process bolsters individual empowerment [11].

The PAR intervention was found to alter the MetS components and psychosocial factors. Moreover, it reduced the MetS-related indices in patients [12] and notably lowered its risk factors, including body weight [8] and BMI [3]. After this intervention, the participants demonstrated enhanced psychosocial factors such as the determination to manage health, self-control [8], empowerment [11], and social support [8]. Although the impact of the PAR on the health-related quality of life has not been validated thus far, it has been reported as a critical psychosocial aspect associated with health in patients with MetS [13]. Particularly, empowerment was indicated as a significant predictor of the community residents’ health status [14]. It refers to the perceived control of multi-level factors related to health promotion [15]. Therefore, to reduce the MetS risk factors in adult women, a participant-centered intervention is needed to maintain healthy behaviors considering the multi-level influencing factors and real-life situations.

Thus far, the MetS-related interventions have addressed health education to prevent MetS and improve its symptoms at the individual and group levels. Counseling for lifestyle improvement, motivational interviewing [16], and supportive programs through group discussions or self-help group activities [7,13] have proven to be effective in enhancing health behaviors, social support [7,12], and metabolic-related indices [13,16]. However, although these interventions temporarily induced changes in perception and health behaviors, there was no lasting effect [17], suggesting that these programs failed to consider that MetS in adult women occurs on multi-levels. Moreover, it revealed the limitation of the researcher-centered approach in persuading participants to solve the problem of cultivating lifestyle habits in daily living.

Therefore, this study aimed to apply the PAR approach in a multi-level context for adult women with the MetS risk factors. The presence of health-related risk factors has been verified in the PAR interventions conducted in healthcare settings to solve health problems [8]. This research attempted to validate the measurements of the individual cognitive elements (e.g., empowerment, health-related quality of life) and determine the PAR intervention’s effects on empowerment, social support, health-related quality of life, metabolic-related indices, and health behaviors.

### 1.2. Purpose of the Study 

This study purported to develop and apply a PAR intervention for adult women with MetS risk factors in communities. Additionally, it aimed to help the participants actively find problems associated with their health behavior improvement, resolve them through lifestyle changes, and assess the aforementioned intervention’s effectiveness.

### 1.3. Hypotheses of the Study 

This research aimed to develop and apply a PAR intervention for adult women with the MetS risk factors in communities:

**Hypothesis** **1** **(H1).** *The experimental group undergoing PAR will have higher empowerment after the intervention as compared to the baseline*.

**Hypothesis** **2** **(H2).** *The experimental group undergoing PAR will demonstrate increased individual-level empowerment post the intervention than at the baseline*.

**Hypothesis** **3** **(H3).** *The experimental group undergoing PAR will have greater community-level empowerment after the intervention as compared to the baseline*.

**Hypothesis** **4** **(H4).** *The experimental group undergoing PAR will have higher social support after the intervention than at the baseline*.

**Hypothesis** **5** **(H5).** *The experimental group undergoing PAR will have an increased health-related quality of life after the intervention as compared to the baseline*.

**Hypothesis** **6** **(H6).** *The experimental group undergoing PAR will have higher physiological metabolism indices after the intervention than at the baseline*.

**Hypothesis** **7** **(H7).** *The experimental group undergoing PAR will have greater health behaviors after the interventionas compared to the baseline*.

## 2. Materials and Methods

### 2.1. Research Design

We examined the effectiveness of the PAR intervention using the one-group pretest-posttest design to verify its influences on empowerment, social support, health-related quality of life, metabolic-related indices, and health behaviors. 

### 2.2. Participants and Ethics

#### 2.2.1. Participants

Regarding participant recruitment, we posted a notice on the community health center’s bulletin board and website. The selection criteria included adult Korean women aged 30 years or above, who were affected by a minimum of one of the five MetS risk factors, which were selected according to the five diagnostic criteria for MetS in Korean adult women: (1) waist circumference ≥85 cm; (2) triglycerides ≥150 mg/dL; (3) HDL cholesterol <50 mg/dL; (4) fasting blood glucose ≥100 mg/dL or higher; and (5) systolic and diastolic blood pressures of ≥130 mmHg and ≥85 mmHg, respectively [18]. MetS is characterized by the clustering of three or more of its risk factors; however, in this study, one or more risk factors were selected for its prevention.

The minimum sample size for a one-tailed paired *t*-test was 41. It was calculated using the G*Power 3.1, with the effect size (f), significance level (α), and power (1-β) set at 0.40, 0.50, and 0.80, respectively. The effect size of this study for calculating is a previous study where middle-aged women improved health behaviors after community based participatory intervention based on a community based participatory research with obesity women [3]. Considering the potential for dropouts, 60 participants were recruited, of which two did not attend the intervention program. Finally, 58 participants were included in the final analysis.

#### 2.2.2. Ethical Considerations 

Prior approval for this study was obtained from the institutional review board (IRB) of the university research institution (IRB No. DIRB-201506-HR-R-014). Each participant’s verbal consent was obtained during the recruitment interview; moreover, the written consent was obtained at a preliminary meeting before the program commenced. They were also informed of anonymity guarantee, compensation, and participation withdrawal at any point without any disadvantages.

### 2.3. PAR Intervention Program Development

**Figure 1:**
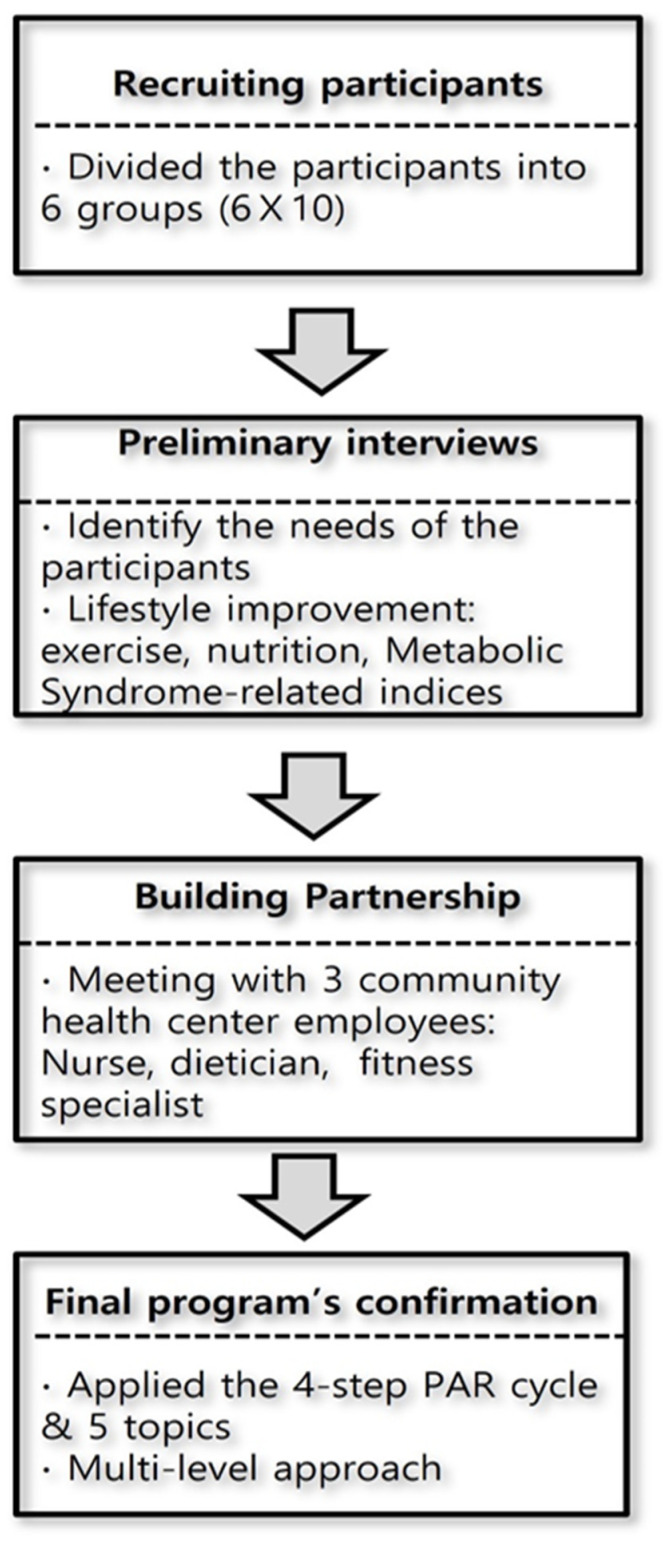
Flow chart of the process of the PAR intervention program’s development.

The PAR intervention program for the adult women with the MetS risk factors was developed in the following stages (Figure 1). Sixty women demonstrating voluntary participation were invited for preliminary interviews in six subgroups (6 × 10) formed according to the program schedule and the intragroup affinity. The topics of interest were discussed considering this study’s purpose, that is, empowering the participants to identify and control their health problems and building a healthcare community that actively shares related experiences with others. The participants were highly interested in exercise and nutrition, and wanted to share ways to manage their general health problems of increased cholesterol, high blood pressure, and obesity. 

The researcher and three community health center employees (a nurse, a dietician, and a fitness specialist) discussed the topics collected by converging the participants’ opinions in the first stage and the methods to apply PAR to the selected topics. The researcher and the participants worked collectively to solve the problems in the PAR process; further, the opinions of the health center employees were fairly reflected. During the discussion, the participants expressed their needs and agreed to include the issues regarding the MetS-related influencing factors that could be applied at the community and individual levels. This resulted in the inclusion of the topic “local environmental improvements for health promotion”.

In this study, a four-step PAR process consisting of look, think, act, and reflect, a modified model of Koch and Kralik’s [10] “Look, Think, Act” cycle in the PAR, was used in conjunction with the multi-level approach (the individual, group, and community levels). The participants were requested to define the problems that impeded the health behaviors in their daily lives (Look), decide and plan the most effective way to improve them (Think), implement the plan (Act), and maintain a diary to reflect on self-motivation and outcomes (Reflect).

#### Final PAR Application

The intervention program of this study was integrated and applied to the four cyclical approaches of PAR to recognize and deal with the factors affecting MetS in a multi-level context (Table 1). The four-step PAR cycle was applied to each of the five topics derived from the preliminary interviews with the participants and the discussion with the community health center staff. This included: (1) health checkup and a health behavior checklist; (2) lifestyle disease, MetS; (3) personalized physical activities; (4) tailored nutrition care; (5) proposals for local environmental improvements for health promotion. Furthermore, the researcher provided the participants with an activity sheet booklet to help them determine and reflect on their health problems.

As part of the PAR cycle’s first step “Look,” the participants were instructed to recognize themselves as individuals affected by the MetS risk factors, make notes about their unhealthy lifestyle habits and health resources on the activity sheet, and examine their health condition. To help them gain a sense of control over their health problems, they were requested to measure their blood pressure, waist circumference, and fasting blood glucose level; additionally, they assessed their health condition by comparing the measured values with the normal ones. They were also encouraged identify common health issues by evaluating the other participants’ measurements, and to expand the scope of their problems from the individual to the community level.

The second step “Think” focused on seeking possible solutions to the health problems identified in the previous stage. Specifically, the activities in this step included thinking about the possible changes in their daily life to resolve their health issues or implement healthy behaviors, as well as identifying the facilitating and impeding factors in health promotion. The participants wrote down the related questions on the activity sheet; they shared their thoughts with the other participants to derive personalized activities. Subsequently, in the third step “Act”, the activities comprised setting concrete personal goals to promote health behavior practices and solve health problems, recording the implementation date, and presenting the weekly checkup plan. All participants were instructed to formulate an action plan according to their schedules and lifestyles, and have it examined by the experts and fellow participants; furthermore, they were encouraged to present their self-motivation to implement the action plan. Finally, in the fourth step “Reflect”, they were requested to self-reflect on the major impeding factors in the health behavior practices’ implementation and plan further action.

To recognize and deal with the multi-level context of the factors affecting the MetS, the individual-level context is to recognize unhealthy lifestyle and health problem. This was based on a previous study by Kang et al. [8] in which bad lifestyle habits and low awareness of diseases were identified as risk factors at the individual-level. The participants’ lifestyle behavior and MetS-related risk factors are identified by creating a checklist-type activity sheet that includes normal and abnormal ranges. In addition, the participants were instructed to frequently check their health status in terms of abdominal circumference, blood pressure, and pulse measurement. At the group-level, social support from family and colleagues was identified as a major influencing factor [7], thus the creation of a support group for those who face the same problems. At the beginning of the program, the most influential participant was selected as the leader, and from the second session onwards, he/she was allowed to act as the leader of the group discussion. To avoid deviating from the topic of discussion, the role of the leader was explained in advance. The support group meeting led to the formation of a self-help group, which would enable them to become the main agents of change in the local community.

According to Hwang et al. [19], at the community-level, it is important to comprehensively understand factors such as the environment and policies of the local community in order to induce a continuous intervention effect. Therefore, in the PAR cycle for each topic, the participants were asked to systematically identify factors from the individual-level to the community-level.

In particular, during the ‘Think’ stage of the PAR cycle which considers the determinants of health, factors affecting MetS are divided into facilitating and hindering factors so that they can think critically. Additionally, in the fifth session, the groups suggested some ideas for community change, and the proposals or ideas that could be put into practice were proposed to the public officers. This PAR cycle and multi-level approach was applied to each of the five PAR sessions.

### 2.4. Instruments

The research instruments consisted of a self-report questionnaire and the diagnostic indices of the MetS.

#### 2.4.1. Empowerment

Empowerment was measured with Perceived Control Scale: Multiple levels of Empowerment Indices developed by Israel et al. [20] and modified and adapted into Korean by Kim et al. [15]. The original 12-item tool contains questions regarding empowerment at the individual (two items), organizational (five items), and community levels (five items). In this study, the organizational level’s five items were excluded for being unfit for the regional characteristics of the research site. The remaining seven items (the individual and community levels) were included for assessment. Each item was rated on a five-point Likert scale (one = strongly disagree, five = strongly agree), wherein a higher score indicated a greater level of empowerment. The Cronbach’s α values, a measure of internal consistency, for the individual- and community-level subscales were 0.66 and 0.63, respectively in Israel et al.’s study [20], 0.82 and 0.87 in Kim et al.’s research [15], and 0.71 and 0.84 in this study.

#### 2.4.2. Social Support

Social support was assessed using the social support scale developed by Abbey, et al. [21], which was translated and validated for the Korean population [22]. The instrument consists of six items which are rated on a five-point Likert scale (one = not at all, five = very much), wherein a higher score indicated a greater level of social support. The Cronbach’s α was 0.77 and 0.89 in the research of Abbey et al. [21] and Jeong and Lee [22], respectively; in this study, it was 0.89.

#### 2.4.3. Health-Related Quality of Life

The health-related quality of life was evaluated using the Euro Quality of Life Five-Dimensional Questionnaire (EQ-5D) developed by the Euro Quality of Life Group and calculated by the Korea Disease Control and Prevention Agency (KDCA) to estimate the quality weights [23]. It consists of five dimensions related to one’s current health state: mobility, self-care, usual activities, pain or discomfort, and anxiety or depression; furthermore, each dimension has three levels of perceived problems (levels 1, 2, and 3: no, some, and extreme problems, respectively). Overall, 243 possible health states can be defined by combining these dimensions and levels. This study employed the weights calculated by the KDCA as follows:

EQ-5D index = 1 − (0.05 + 0.096 × M2 + 0.418 × M3 + 0.046 × SC2 + 0.136 × SC3 + 0.051 × UA2 + 0.208 × UA3 + 0.037 × PD2 + 0.151 × PD3 + 0.043 × AD2 + 0.158 × AD3)

where M: mobility, SC: self-care, UA: usual activities, PD: pain/discomfort, AD: anxiety/depression.

#### 2.4.4. Metabolic-Related Indices

The metabolic-related indices were assessed according to the MetS diagnostic criteria. Five tests were performed to examine waist circumference, triglycerides, HDL cholesterol, fasting blood glucose, and blood pressure. These factors, along with the BMI, were measured before and after the study (baseline and post-intervention). The waist circumference was evaluated by placing a tape measure horizontally between the coastal margin and the iliac crest. For triglycerides, HDL cholesterol, and fasting blood glucose, venous blood was collected after overnight fasting (12 h). A research assistant measured the systolic and diastolic blood pressures twice using a manual sphygmomanometer; additionally, the average of the two measurements 30 min apart was calculated. The BMI was determined using a body composition analyzer (Inbody770, Biospace, Seoul, Korea).

#### 2.4.5. Health Behavior

Health behavior included variables such as physical activity, reading nutrition labels, and drinking alcohol that were self-reported. High (vigorous), moderate, and low intensity (walking) physical activities were examined. High-intensity physical activity refers to breathing considerably heavily than normal and experiencing a large increase in the usual heart rate for 20 min or more at a time for over thrice a week. Furthermore, the moderate and low intensity physical activities denote the case of breathing slightly harder than normal for 30 min or more or walking 5 days weekly. Nutrition entails the reading of nutrition labels when purchasing processed foods. Additionally, alcohol consumption was measured by the monthly drinking experience; high-risk alcohol consumption was defined as drinking five or more glasses of alcohol daily, twice a week. Smoking was excluded from the post-test because there were no smokers in the pre-test.

### 2.5. Data Collection

The data were collected from May to November 2016 based on the following procedure.

#### 2.5.1. Preparation by the Researcher and the Training of the Research Assistants

The researcher (author) has served as the community health center’s health promotion program developer and instructor as well as an advisory member of the Integrated Health Promotion Support Group of U Metropolitan City since 2010. As part of this study’s preparations, the researcher held four meetings over two months with the community health center’s working staff (a nurse, a dietician, and a fitness specialist) to discuss the implementation of the PAR intervention program. The staff conducted two or three out of the five sessions per group after two training sessions based on the PAR program operation guidelines written by the researcher. Additionally, they facilitated the PAR implementation through a weekly research meeting to share their observations and provide feedback.

#### 2.5.2. Pre-Test

The pre-test was obtained at an assessment meeting for each of the six subgroups, followed by body measurements, blood tests, and questionnaire surveys. The examination of the metabolic indices was subjected to body measurements and blood tests after 12 h of overnight fasting. The blood samples were analyzed in the community health center laboratory. Further, the participants’ blind test samples were labeled using serial numbers to protect their personal information, followed by a transfer within six hours for an immediate blood analysis.

#### 2.5.3. Experimental Treatment

The final PAR program was applied to six groups of ten people each weekly for five sessions. It aimed at reducing the MetS risk factors according to the predetermined concrete goal, core contents, and details of the joint PAR activities involving the researcher and the participants. The individual-level approach enabled the participants to practice healthy behaviors in the context of daily living according to their level of risk awareness of diseases through weekly private phone consultations. The group-level method helped them perform such behaviors with the aid of a 10-member group and experts in each session. Moreover, the community-level approach considered their needs expressed in the preliminary meetings to induce active participation in the program. Starting from the second session, the participants wrote down example sentences in the activity sheet during the group discussion in the order, for the health determinants to be recognized and expanded to the community level. In the last session, the participants evaluated their participation in the PAR, provided feedback, and presented their plans. Experts from various fields also participated in the program as partners. In addition to the researcher, one nurse, one dietician, and one fitness specialist from the community health center where the program was conducted participated as partners too. They provided information on their area of specialization and helped individual participants plan and implement daily exercise, nutrition, and healthy lifestyle practices tailored to their life.

Each session began with a 30-min preparatory activity to assess the health status. The activity sheet provided in advance, subsequent to which the assessment for health status, the MetS self-diagnosis, exercise compliance, eating habits, and health resources stock-taking were performed in sessions 1, 2, 3, 4, and 5, respectively. The participants maintained a checklist and a reflection diary session 2 onward to share difficulties and experiences regarding enhancing compliance with healthy life practices. In the 40-min main session, the participants completed the activity sheet regarding the impeding factors related to the session’s topic to contemplate about the methods necessary to solve the identified health problems. Additionally, topic-related information was provided through booklets, audiovisual materials, and demonstrations to raise awareness regarding the health issues, followed by a group discussion about perceptions of them. In session 2, a group leader was selected among the participants to lead the conversation and to motivate the participants toward a more dedicated involvement. In a 20-min closing session, they committed themselves to action by writing down their goals as well as creating an action plan and an activity sheet. They were supported by the researcher or a research assistant through weekly phone consultations to identify and tackle problems related to health behavior practices.

#### 2.5.4. Post-Test

The post-test (post-intervention measurements) was conducted after completing the final PAR session of the six subgroups as the pre-test.

### 2.6. Data Analysis

The statistical data analysis was performed using the SPSS 25.0 program (IBM Corp., Armonk, NY, USA). The demographic characteristics were analyzed using descriptive statistics. Furthermore, the normality tests of the dependent variables were performed using the Shapiro-Wilk test. A paired t-test was conducted to compare the pre- and post-intervention survey data, psychosocial measurements, and MetS-related indices. The McNemar test was employed to compare the pre- and post-intervention health behaviors.

## 3. Results

### 3.1. Demographic and Metabolic-Related Characteristics of the Study Participants

The mean age of the participants (*n* = 58) was 45.81 years. The most numerous age group was the 50s, and older was 36.2%. Over half (77.6%) of the participants followed a religion; further, 51.7% were high school graduates or lower. The most frequent monthly income range was three to five million won (55.2%), and the majority of the participants were unemployed (87.9%; Table 2). Of the 58 participants, 37 (63.8%) had a BMI of 25 or over. Additionally, 12 (20.7%) and 5 (8.6%) participants had hypertension and diabetes, respectively. Most participants had MetS (69.0%); moreover, 31.0% of them had one or two metabolic risk factors.

### 3.2. Effects of the PAR Program

As a result of the intervention, compared to the pre-test, the participants demonstrated significantly improved empowerment scores at both individual (t = −3.36, *p* = 0.001) and community levels (t = −4.71, *p* < 0.001). Similarly, they had significantly enhanced social support (t = −3.27, *p* = 0.002) and health-related quality of life (t = −3.16, *p* = 0.003) (Table 3).

Furthermore, as compared to the pre-test, the participants reported significant improvements in six out of the eight metabolic-related indices, including waist circumference (t = 7.02, *p* < 0.001), fasting blood glucose (t = 2.57, *p* = 0.013), systolic blood pressure (t = 3.27, *p* = 0.002), diastolic blood pressure (t = 3.43, *p* = 0.001), total cholesterol (t = 2.40, *p* = 0.020), and BMI (t = 4.50, *p* < 0.001) after partaking in the PAR program (Table 3).

Significant pre- and post-test intervention differences were found in the high- (*p* < 0.001) and moderate-intensity physical activities (*p* < 0.001) as well as the reading of nutrition labels (*p* < 0.001; Table 4).

## 4. Discussion

### 4.1. Psychosocial Factors

This study developed a five-week PAR program for improving health behaviors in the community-based adult women with MetS risk factors; further, its influences were verified. The PAR program demonstrated statistically significant effects in improving empowerment, social support, health-related quality of life, and certain metabolic-related indices.

As compared to the pre-test, the post-intervention empowerment score improved significantly. Thus far, no prior research had directly demonstrated the change in empowerment resulting from the PAR application. However, an intervention with a reinforced empowerment strategy had reportedly improved the MetS risk factors and self-management behaviors in hypertensive patients with MetS [12]. Empowerment is perceived regulation at multiple levels in health promotion [15]; it is manifested as control over the factors affecting people’s lives [20] and evaluation of the individual impact in achieving the common goal by defining problems, mobilizing resources, formulating strategies, and implementing projects in the community context [24]. In this study, the increased empowerment was verified with the participants’ improved control over health-affecting factors and the evaluation of their influence in the community. This was supported by Hwang and Kim’s [8] study, wherein the PAR enhanced the health management will and the self-regulation ability of the workers in small workplaces; similarly, Taylor et al. [25] reported the PAR’s effectiveness in strengthening smokers’ motivation and willpower to quit smoking. In this study, empowerment for the self-management of health may have been reinforced in iterating the multi-level approach. This includes the individual (information provision, customized counseling), group (group support), and community levels (active-participation, researcher-participant partnership), as well as the four-step PAR cycle approach at each session. It involved experiencing success and failure in complying with health behaviors and repeatedly conducting self-reflection by utilizing the individual or community resources. However, self-reported empowerment has limitations in understanding its multi-level, dynamic concept that may be ensured by assessing the actual control and influence [24]. In particular, the community-level empowerment requires a qualitative approach to understand the outcomes effectively, such as the context and the mechanism of influencing change regarding exercise in the community, improvement of the situation and policy participation, and political voice for the community empowerment to influence the local community [24]. Therefore, it is necessary to examine the PAR program participants’ multi-level empowerment change process in a follow-up qualitative study.

Social support for the adult women with the MetS risk factors indicated significant improvement after the PAR intervention. This is consistent with the previous research’s findings, that social support improved for these women after participating in a coherence enhancement [13] and a self-care reinforcement program [7]. However, this was inconsistent with a research that reported no change in workers’ social support after the PAR application [8]. In this study, the primary approach to social support was the help obtained by engaging in group activities with the other members affected by similar problems and having cooperative relationships with the experts. In group activities, the participants shared their experiences of identifying and solving problems related to their lifestyles, recognizing helpful and harmful health resources, and changing health behaviors. Furthermore, the community health center members, who were health behavior specialists (the nurse, the dietician, and the fitness specialist), continuously examined the target achievement level and provided counseling, guidance, and feedback on the health behavior practices. This provided constant encouragement for compliance and motivation for continuous practice. Such professional intervention allowed the participants to realize, through conversations and discussions, that their problems were not specific, but rather common to all; this helped them overcome their issues by observing the members showing positive changes in their interactions [7]. A particular significance of this study is that it quantitatively verified the effect of social support in the PAR by using a psychometric tool.

The health-related quality of life of the adult women with MetS risk factors improved significantly in the experimental group after the PAR application. Although a different tool was used, this finding is partially consistent with the study wherein a lifestyle intervention based on an integrated model of knowledge, skill, and behavior enhanced the physical health-related quality of life of the women with MetS [13]. The health-related quality of life is an essential factor related to the health status, evaluated based on personal experiences, beliefs, expectations, and perceptions of physical, mental, and social well-being [26]. The validity of the health-related quality of life scale employed in this research was examined not only in a specific population group such as patients, but also in the general one. It is a widely utilized instrument in the healthcare sector for its ability to compare diverse disease-related outcomes [23]. People with a greater sense of empowerment and social involvement perceive their health status to be higher, have better health behaviors such as physical activity and exercise, and report a greater health-related quality of life [15,27]. These results support that the increased in empowerment and social support after PAR intervention may have improved the health-related quality of life.

### 4.2. Metabolic-Related Indices

The metabolic-related indices of the adult women with the MetS risk factors improved after the PAR application, as compared with the baseline values. The waist circumference decreased by 2.15 cm after the PAR intervention than the baseline value, which is closer to the 85 cm threshold value set for the Korean women considering their demographic characteristics and cultural factors. Due to weight loss, their BMI also decreased from 26.24 (pre-test) to 25.60 (post-test). Abdominal obesity further increases the prevalence of metabolic disorders [28], wherein abdominal fat-induced insulin resistance and impaired glucose tolerance increase the risk of MetS through an elevated blood glucose [29]. Visceral obesity is an independent predictor of insulin sensitivity and impaired glucose tolerance. Further, chronic hyperglycemia causes oxidative stress in tissues, thus causing complications in patients with diabetes [30]. According to a research by Kim [3], the body fat mass and the visceral fat area were significantly reduced after the PAR intervention. In this study, the fasting blood sugar decreased by 5.05 mg/dL after the intervention as an outcome of the improved abdominal circumference and BMI. The systolic and diastolic blood pressures improved post-intervention. The Korean women with MetS have reported insufficient calcium and excessive sodium intake [31] that increases their blood pressure [32]. The adult women who participated in this study’s PAR program learned nutrition management for MetS prevention and compared it with their usual diet. They improved their eating habits and reduced their intake of high-sodium foods, such as soups. Their concrete health behavior plan and practice integrated into their lifestyle contributed to decreasing their blood pressure.

In this study, MetS-related indicators changed statistically except for HDL cholesterol, while there is room for consideration in the clinical significance of effect size (especially BMI = 0.64 kg/m^2^). In a study that applied educator-centered health promotion programs (five sessions) to middle-aged women in the community [33], the abdominal circumference decreased to 1.11 cm, systolic blood pressure decreased to 3.58 mmHg, and the diastolic blood pressure decreased to 2.51 mmHg, while BMI and blood sugar were not statistically significant in the post-test compared to the pre-test. These results suggest that, although the intervention period is relatively short, the participant-centered PAR study is effective in changing MetS-related indices.

The unsuccessful reduction of the HDL cholesterol may be attributable to an insufficient exercise intensity and a short intervention period. Given that “the National Cholesterol Education Program—Adult Treatment Panel III” recommends an intervention period of six months or longer for patients with MetS, it is suggested that a follow-up study investigate the relationship between the intensity of physical activity and the metabolic parameters. Additionally, it should apply a more extended intervention period to induce changes in the HDL cholesterol values.

### 4.3. Health Behaviors

Regarding health behaviors after applying the PAR, vigorous activity, moderate-intensity physical activity, and the reading of nutrition labels improved. This is in line with the findings of Hwang and Kim [8], that after implementing the PAR on labor workers, the practice level of physical activity and diet enhanced. This is also similar to the study by Kim [3] that applied the PAR to obese middle-aged women in the local community, thus improving the practice level of physical activity and the dietary guidelines. This enhancement was potentially affected by the PAR process in which the subjects examine their health status, formulate an action plan, set specific goals in daily life, practice the action, and reflect on their experiences. The health promotion programs for public health have been mainly developed in the researcher-centered studies based on a natural science paradigm utilizing standardized tools with the purpose of identifying and applying precise laws to explain, predict, and control a phenomenon [34]. Existing researcher-centered programs have been carried out on the areas in Korea that comprise a healthy lifestyle, such as nutrition, exercise, anti-alcoholism, and smoking cessation. The program operation was also conducted through unilateral demonstrations by experts [3]. The PAR is a participant-centered intervention; it is a method of finding and implementing health behavior improvements through a reflective practice in the context of one’s own life. Moreover, it has been shown that health behaviors persist even after the intervention [8]. Thus, to prevent and manage MetS, where a consistent lifestyle improvement is important, the PAR approach that can lead to practical action plans in the context of the subject’s life seems to be effective. According to a study evaluating the persistence of PAR health behaviors [8], risk perception, social support, and self-regulation of the disease were found to be significant factors. In this study, the risk perception of MetS was strengthened by identifying the factors affecting MetS at the individual, group, and community levels, while social support and empowerment at the individual and community level were improved through the intervention of participant-centered group meetings. Thus, it is expected that this effect will persist even after the intervention is finished.

### 4.4. Limitations

This study has certain limitations. First, it investigated the effects of applying the PAR to a group of adult women with the MetS risk factors in only one community health center in a South Korean city. Therefore, the results cannot be generalized. Second, the researcher who played a role in promoting the inter-group dynamics in the participant-centered intervention did not partake in the overall program. In the PAR approach, the application of participant-centered interventions and the contextual factors that occur during the intervention process must be interpreted precisely. Thus, the limited participation of the researcher in this study may have led to errors when interpreting the outcomes. However, through the pre-meeting and the intervention guidelines, the study attempted to ensure the intervention’s consistency by training the participant-centered program for participants and encouraging them to contribute in the program organized by the researcher. Finally, the intervention period of five weeks was insufficient to assess the long-term changes (e.g., HDL cholesterol). Therefore, it is suggested the impact of the PAR intervention be verified in the follow-up studies with longer intervention periods and randomization.

## 5. Conclusions

In this study, a PAR program was developed to improve the health behaviors of adult women with MetS risk factors, and its effects on improving empowerment, social support, health-related quality of life, metabolic-related indices, and health behaviors, were verified. The researcher investigated the educational needs and the program operation preferences of the participants. As a result of the PAR application, empowerment, social support, and health-related quality of life improved significantly in the post-test, as compared with the pre-test. The post-intervention values of the metabolic-related indices (except the HDL) and the health behaviors (including physical activity and reading nutrition labels) increased significantly in the pre-test. These results are significant in that the PAR effectively enhanced empowerment, social support, health-related quality of life, and health behaviors; further, it provided the primary data for improving the metabolic-related indices. Consequently, it is suggested that the PAR program developed in this study be applied to adult women in diverse living contexts using a longitudinal study and a randomized control design to assess its long-term intervention effects on the metabolic-related indices.

## Figures and Tables

**Table 1:** Contents of the PAR program.

Session	Topic	Details	PAR Cycle	Multi-Level Context
1	Health checkup and health behavior checklist	Health self-assessment Understanding and devising strategies for health behavior Sharing experiences of health behavior Identification of facilitators and inhibitors in health promotion Planning and implementing health behavior strategies	Look: Look at your health status: vital signs (blood pressure, heart rate), body mass index (height, weight), waist circumference, healthy behavior practice checklist. Think: Think about changes to improve health behaviors as well as the facilitators and inhibitors in health promotion. Act: Plan healthy behaviors and commit yourself to implementing them. Reflect: Reflect on your actions and outcomes through a reflection diary and plan further action.	Individual Group Community
2	Lifestyle disease, MetS	Examining the MetS risk factors Understanding lifestyle diseases Identification of facilitators and inhibitors in the MetS management Planning and implementing strategies for the MetS prevention	Look: Look at your MetS risk factors. Think: Think about changes to prevent MetS as well as the facilitators and inhibitors of your management of MetS risk factors. Act: Plan healthy behaviors and commit yourself to implementing them. Reflect: Reflect on your actions and outcomes through a reflection diary and plan further action.	Individual Group Community
3	Personalized physical activities	Assessing physical activity Comprehending and selecting appropriate physical activity types Recognition of facilitators and inhibitors in physical activity Planning and implementing strategies for physical activity	Look: Look at your physical activities (compliance checklist). Think: Think about changes to improve your compliance with an exercise plan as well as related facilitators and inhibitors. Act: Plan healthy behaviors and commit yourself to implementing them. Reflect: Reflect on your actions and outcomes through a reflection diary and plan further action.	Individual Group Community
4	Personalized nutrition care	Evaluating the nutrition state Understanding nutrition management Identification of facilitators and inhibitors in nutrition management Planning and implementing nutrition care	Look: Look at your eating habits. Think: Think about changes to improve your nutritional intake as well as the related facilitators and inhibitors. Act: Plan healthy behaviors and commit yourself to implementing them. Reflect: Reflect on your actions and outcomes through a reflection diary and plan further action.	Individual Group Community
5	Proposals for local environment improvement for health promotion	Assessing health resources (at the family, community level) Comprehending health resources Identification of facilitators and inhibitors in the health resources’ utilization Proposing strategies for improving community health environment	Look: Look at your health resources. Think: Think about changes to improve the utilization of health resources as well as the related facilitators and inhibitors. Act: Plan healthy behaviors and commit yourself to implementing them. Reflect: Reflect on your actions and outcomes through a reflection diary and plan further action.	Individual Group Community

PAR = participatory action research; MetS = metabolic syndrome.

**Table 2:** Demographic and metabolic-related characteristics of the study population (*N* = 58).

Characteristics	Categories	*n* (%)
Age (in years)	30 to <40	18 (31.0)
	40 to <50	19 (32.8)
	≥50	21 (36.2)
	Mean ± SD	45.81 ± 9.13
Religion	Yes	45 (77.6)
	No	13 (22.4)
Education	High school	30 (51.7)
	College	28 (48.3)
Income (KRW/month)	<3,000,000	15 (25.9)
	3,000,000 to <5,000,000	32 (55.2)
	≥5,000,000	11 (19.0)
Job	Yes	7 (12.1)
	No	51 (87.9)
BMI	18.5~22.9 kg/m^2^	8 (13.8)
	23~24.9 kg/m^2^	13 (22.4)
	≥25 kg/m^2^	37 (63.8%)
Hypertension	Yes	12 (20.7)
	No	46 (79.3)
Diabetes	Yes	5(8.6)
	No	53 (91.4)
Metabolic risk factors	1 or 2	18 (31.0)
	≥3	40 (69.0)

SD = standard deviation.

**Table 3:** Effects of the PAR program on the psychosocial factors and the metabolic-related indices (*N* = 58).

Variables	Pre-Test	Post-Test	t (*p*)
	Mean (SD)	Mean (SD)	
Empowerment			
Individual-level	3.26 (0.66)	3.72 (0.70)	−3.36 (0.001)
Community-level	3.07 (0.59)	3.50 (0.41)	−4.71 (<0.001)
Social support	3.76 (0.51)	4.03 (0.35)	−3.27 (0.002)
Health-related QoL	0.87 (0.13)	0.92 (0.04)	−3.16 (0.003)
Waist circumference (cm)	88.09 (8.64)	85.94 (7.80)	7.02 (<0.001)
HDL cholesterol (dL)	56.91 (15.74)	57.49 (11.85)	−0.62 (0.535)
Fasting blood glucose (mg/dL)	109.31 (20.03)	104.26 (15.31)	2.57 (0.013)
Systolic blood pressure (mmHg)	128.12 (19.17)	122.88 (17.74)	3.27 (0.002)
Diastolic blood pressure (mmHg)	83.38 (11.55)	80.09 (8.80)	3.43 (0.001)
Body mass index (kg/m^2^)	26.24 (3.54)	25.60 (3.32)	4.50 (<0.001)

SD = standard deviation; QoL = quality of life; LDL = low-density lipoprotein; HDL = high-density lipoprotein.

**Table 4:** Effects of the PAR program on health behaviors (*N* = 58).

Variables	Categories	Pre-Test	Post-Test	McNemar’s *p*
		*n* (%)	*n* (%)	
Physical activity				
High-intensity	No	58 (100.0)	41 (70.7)	<0.001
	Yes	-	17 (29.3)	
Moderate-intensity	No	54 (93.1)	51 (87.9)	<0.001
	Yes	4 (6.9)	7 (12.1)	
Low-intensity	No	22 (37.9)	14 (24.1)	0.115
	Yes	36 (62.1))	44 (75.9)	
Reading of the nutrition labels	No	37 (63.8)	5 (8.6)	<0.001
	Yes	21 (36.2)	53 (91.4)	
Alcohol consumption	No	17 (29.3)	12 (20.7)	0.332
	Yes	41 (70.7)	46 (79.3)	
High risk alcohol consumption *	No	55 (94.8)	46 (100.0)	0.250
(N = 46)	Yes	3 (5.2)	-	

* Number of alcohol consumption ‘yes’ responses.

## Data Availability

The data is available upon request due to ethical and privacy restrictions.
